# Effects of silver nanocolloids on plant complex type *N*-glycans in *Oryza sativa* roots

**DOI:** 10.1038/s41598-018-19474-z

**Published:** 2018-01-17

**Authors:** Risa Horiuchi, Yukari Nakajima, Shosaku Kashiwada, Nobumitsu Miyanishi

**Affiliations:** 1 Graduate School of Life Sciences, Toyo University, Gunma, 374-0193 Japan; 2 Department of Life Sciences, Toyo University, Gunma, 374-0193 Japan; 3 Research Centre for Life and Environmental Sciences, Toyo University, Gunma, 374-0193 Japan; 4 Graduate School of Food and Nutritional Sciences, Toyo University, Gunma, 374-0193 Japan

## Abstract

Silver nanomaterials have been mainly developed as antibacterial healthcare products worldwide, because of their antibacterial activity. However, there is little data regarding the potential risks and effects of large amounts of silver nanomaterials on plants. In contrast, *N*-glycans play important roles in various biological phenomena, and their structures and expressions are sensitive to ambient environmental changes. Therefore, to assesse the effects of silver nanomaterials, we focused on the correlation between *N*-glycans and the effects of silver nanomaterials in plants and analyzed *N*-glycan structures in *Oryza sativa* seedlings exposed to silver nanocolloids (SNCs). The phenotype analysis showed that the shoot was not affected by any SNC concentrations, whereas the high SNC exposed root was seriously damaged. Therefore, we performed comparative *N*-glycan analysis of roots. As a result, five of total *N*-glycans were significantly increased in SNC exposed roots, of which one was a free-*N*-glycan with one beta-*N*-acetylglucosamine residue at the reducing end. Our results suggest that the transition of plant complex type *N*-glycans, including free-*N*-glycans, was caused by abnormalities in *O. sativa* development, and free-*N*-glycan itself has an important role in plant development. This study originally adapted glycome transition analysis to environmental toxicology and proposed a new category called “Environmental glycobiology”.

## Introduction

Nanomaterial is a general term for small substances that are 1–100 nm in diameter. Nanomaterials have many unique electrical, chemical, and physical properties and are used in electronics, medicine, and healthcare fields. Silver nanomaterials have been developed and mainly used for their antibacterial activities in clothes, appliances, cosmetics, and plastics. However, there have been concerns that silver nanomaterials are likely released into the aquatic environment through factory and household wastewater on a large scale, and there are also concerns regarding the effects of silver nanomaterials on aquatic organisms and ecological systems. The toxicity of silver nanomaterials in the embryos of aquatic organisms such as medaka and zebrafish has been reported^1,2^. The toxicity affected the expression of morphogenesis- and cell proliferation-related genes and induced the increase of severe development abnormalities and mortality. The toxicity of silver nanomaterials was dependent on the particle size, shape, and capping materials^3,4^. Toxicity of silver nanomaterials has also been reported in plants, with the toxicity affecting germination, development, and photosynthetic efficiency because of the induction of oxidative stress, cytotoxicity, and genotoxicity^5,6^. For example, silver nanoparticle exposure significantly reduced root elongation, shoot and root fresh weights, and total chlorophyll and carotenoid contents^7^. Colman *et al*. showed that low silver nanoparticle concentrations caused a decrease in biomass^8^. Furthermore, the toxicity of silver nanoparticles affects the expression of several proteins that are mainly involved in primary metabolism and cell defense in wheat seedlings^9^. At a gene level, silver nanoparticles activate gene expression involved in plant cellular events, including cell proliferation, metabolism, and hormone signaling pathways^10^. The above-mentioned studies showed that silver nanomaterials have high toxicity in plants. Therefore, the risk assessment of silver nanomaterials in plants is important.

Asparagine (N)-linked glycans (*N*-glycans) are comprised several types of monosaccharides, forming complex compositions and linkage types. *N*-Glycan has a trimannosyl core structure [Man alpha1–6(Man alpha1-3)Man beta1-4GlcNAc beta1-4GlcNAc-Asn], which is a common feature in eukaryotes. Many *N*-glycan structures are linked to proteins or peptides and are closely involved in all life phenomena, such as development, signaling, and cell-to-cell recognition. In plants, *N*-glycan structures are categorized into three main types: high-mannose, complex, and paucimannose types; except for hybrid types. A characteristic of plant-specific *N*-glycans is the addition of beta1,2-xylose and alpha1,3-fucose to the trimannosyl core structure. High-mannose type *N*-glycans are synthesized in endoplasmic reticulum (ER), and other type *N*-glycans are synthesized in the Golgi apparatus. Paucimannose type *N*-glycans are linked to vacuole proteins and complex type *N*-glycans are linked to secretory proteins. In addition, cell alterations are reflected in gene expressions through cell signaling, whereas *N*-glycan is synthesized as a result of the integral expression of glycosyltransferase genes, and *N*-glycan structure is sensitive to slight environmental changes^11^. Therefore, *N*-glycan structural analysis is valuable for the risk assessment of silver nanomaterial toxicity in plants. However, there is little data regarding the toxicity of silver nanomaterials in glycobiology. In this study, to assesse the effects of silver nanomaterials, we focused on the correlation of *N*-glycan structures and the effect of silver nanomaterials in *Oryza sativa* and analyzed the *N*-glycan structures in SNC exposed *O. sativa* seedlings.

## Results and Discussion

### Phenotype analysis of *O. sativa* seedling exposed to silver nanocolloids (SNCs)

**Figure 1:**
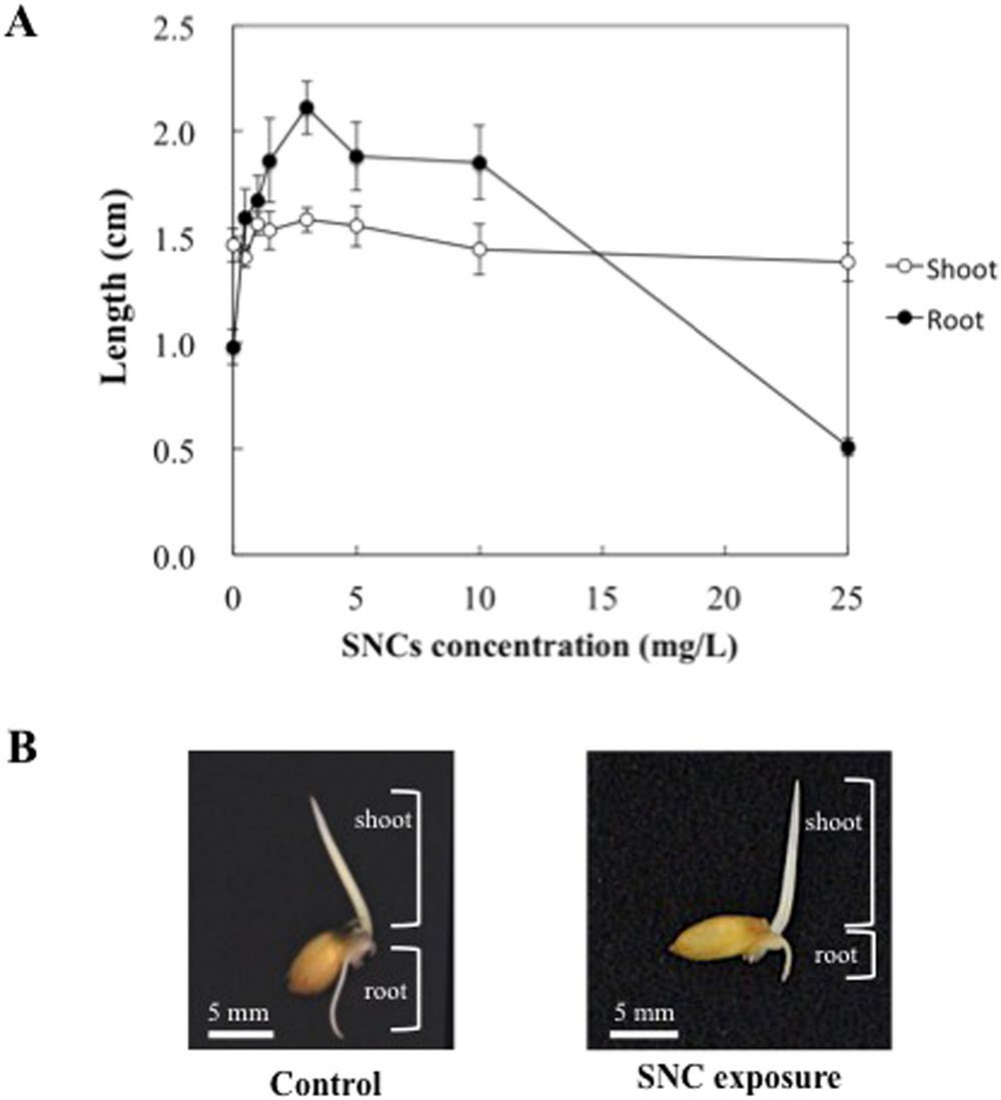
Phenotype analysis of *O. sativa* seedling exposed by SNCs. (**A**) Length of shoots and roots, opened circles indicated shoot and closed circles indicated root. Error bars represent ± one standard deviation from the mean of 20 replicates. (**B**) Overall phenotypes of *O. sativa* seedling.

To observe the effect of SNCs on *O. sativa* seedlings, *O. sativa* seeds were grown with and without SNC exposure. Germination rate was 95% (control), 100% (SNCs 0.5 mg/L), 100% (SNCs 1.0 mg/L), 95% (SNCs 1.5 mg/L), 100% (SNCs 3.0 mg/L), 95% (SNCs 5.0 mg/L), 90% (SNCs 10 mg/L), 100% (SNCs 25 mg/L) after 48 h incubation. The result shows that there is no effect on germination rate at any concentration of SNC for 48 h exposure in *O. sativa*. Figure 1A shows the results of root and shoot elongation in *O. sativa* exposed to SNCs at 0 (control), 0.5, 1.0, 1.5, 3.0, 5.0, 10, and 25 mg/L for 96 h. Shoot and root length of control was 1.46 ± 0.08 cm and 0.98 ± 0.08 cm, respectively. The shoot elongation was not affected at any SNC concentration, whereas the root length increased from 0.5 to 10 mg/L based on Fig. 1A; however, in roots exposed to 25 mg/L SNCs, the lengths were two times lower than those of the control. Representative images of control and 25 mg/L SNC exposed *O. sativa* seedlings are shown in Fig. 1B. From the phenotype analysis, root length was seriously affected by 25 mg/L of SNC exposure.

The effect of SNCs was also observed in other plants. SNCs also have a significant effect on *Arabidopsis thaliana* and poplar development^12^. SNCs are mainly present in two forms: silver nanoparticles and free ions (Ag^+^), which are derived from silver nanoparticles. Free Ag^+^ is more poisonous than SNCs because of its oxidative potency. Wang *et al*.^12^ demonstrated that free Ag^+^ tends to accumulate in *A. thaliana* roots. Previous studies also showed that silver nanoparticles or free Ag^+^ inhibited the growth of *O. sativa* roots^13^, and these materials affect cell metabolism-related proteins^14^. In addition, the effects of SNCs or free Ag^+^ occur in ER- and vacuole-localized proteins of *Eruca sativa*^15^. Nair *et al*. reported that total sugar levels are decreased in SNC exposed *O. sativa* seedlings^7^. From these reports and our observation, SNCs and SNC derived molecules may affect *N*-glycans that are synthesized in the ER and Golgi apparatus of *O. sativa* roots. Therefore, we focused on the correlation of *N*-glycan structures and silver nanomaterials in *O. sativa* and analyzed the *N*-glycan structures in SNC exposed *O. sativa* roots.

### *N*-Glycan analysis of SNC exposed *O. sativa* roots

**Figure 2:**
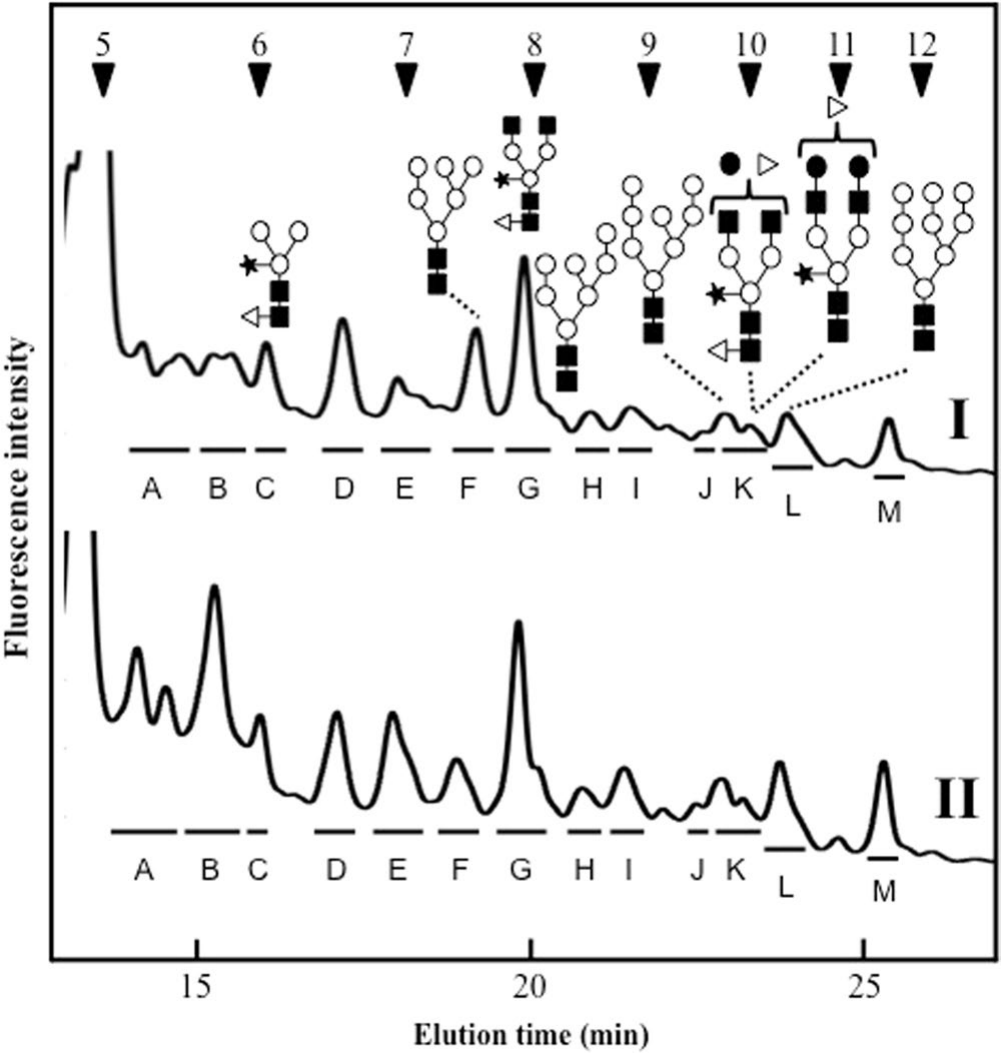
Results of size-fractionation HPLC analysis of PA-*N*-glycans derived from *O. sativa* seedlings. (I) Control, (II) SNCs exposure, PA-*N*-glycans were applied to a Cosmosil 5NH_2_-MS column (4.6 ID × 150 mm). Arrowheads 5–12 indicate the degree of polymerization of PA-isomaltooligomer. The opened circle, closed square, opened triangle, closed star, closed circle represent mannose, *N*-acetylglucosamine, fucose, xylose, and galactose residues, respectively.

**Figure 3:**
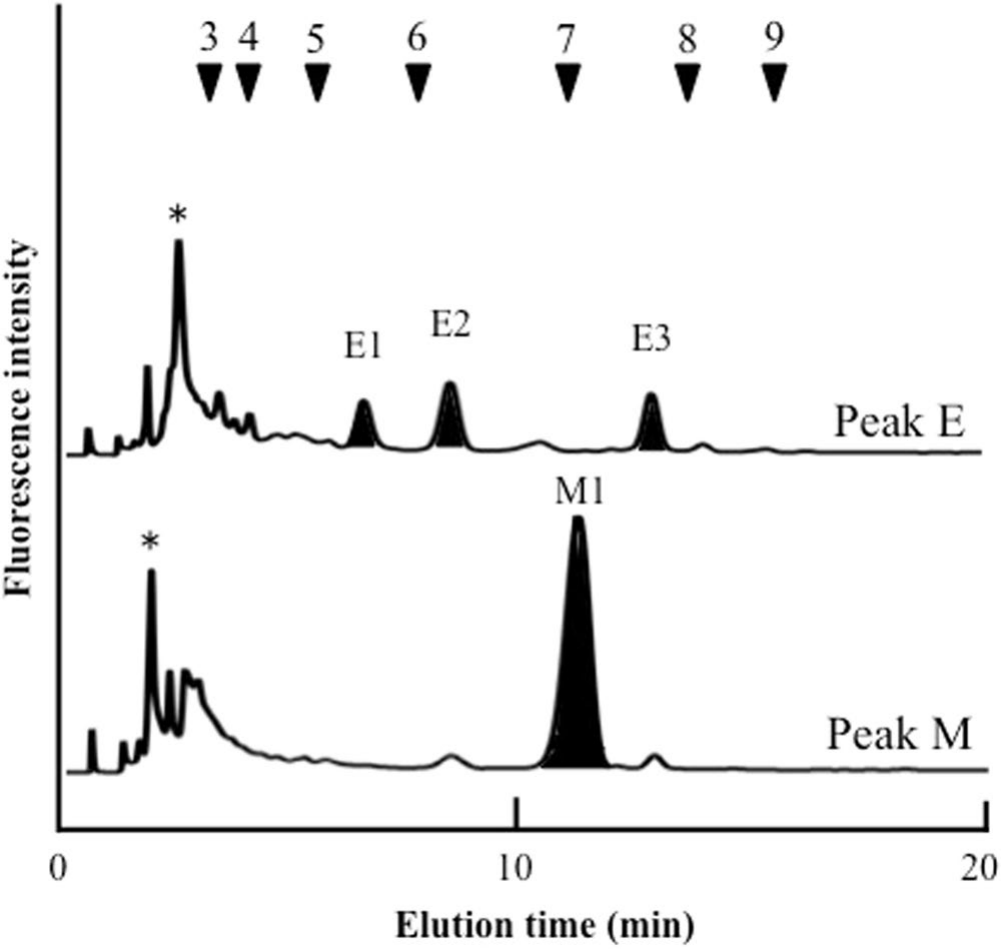
Result of reversed phase HPLC analysis of peaks E and M. The closed areas indicated elution positions of known-*N*-glycan. The peaks marked by the asterisks indicated non-specific peaks.

*N*-Glycans were prepared by hydrazinolysis, *N*-acetylation, and pyridylamination (PA). The resulting PA-*N*-glycans were separated according to their degree of saccharide polymerization by size-fractionation HPLC. Then, *N*-glycans of control and 25 mg/L of SNC exposed roots were compared (Fig. 2I, control and II, SNC treatments). Thirteen peaks were detected (indicated by bars). The areas of peaks B, E, and M increased in SNC exposed roots, and in particular, peak B was extremely increased in SNC exposed roots. Therefore, to identify each *N*-glycan structure in detail, reversed phase HPLC was performed, and branched *N*-glycan isomers were separated. Reversed phase HPLC analysis revealed three major peaks (peaks E1, E2, and E3) of peak E (Fig. 3). Comparing the HPLC elution times with known-position *N*-glycans, peaks E1, E2, and E3 coincided with those of ^GN^M3FX, _GN_M3FX, and GN2M3X, respectively. Similarly, one major peak, M1, was detected in reversed phase HPLC analysis, and peak M1 coincided with the elution position of Gal2F2GN2M3FX.

Table 1 shows the identified *N*-glycan structures and ratios derived from peaks E1, E2, E3, and M1, and each *N*-glycan is shown in terms of percentage proportion relative to GN2M3FX (peak G). The largest *N*-glycan Gal2F2GN2M3FX was increased three fold after SNC exposure. For other plant complex type *N*-glycans, ^GN^M3FX, _GN_M3FX, and GN2M3X, SNC exposure caused up to five- or six-fold higher accumulations than the control. In general, complex type *N*-glycans are formed in from the *cis*-Golgi to *medial* Golgi apparatus, and higher complex modifications occur downstream in the synthetic pathway. Recent reports showed that protein-linked complex type *N*-glycans are related to proper targeting and functioning of linking proteins^16,17^ and also showed that complex type *N*-glycans play an important role in resistance to external stresses such as salt stress^11,18,19^. These reports showed that the Golgi-localized *N*-glycan synthetic enzymes are related to plant growth and development, and their defect inhibited growth and caused abnormalities. Therefore, the transition of complex type *N*-glycans may be related to the disorder of Golgi-localized *N*-glycan synthetic enzymes and genes in SNC exposed roots. The relatively small *N*-glycans ^GN^M3FX, _GN_M3FX, and GN2M3X were affected by SNCs more significantly. These results imply that the upstream part of the *N*-glycan complex pathway was affected by SNCs; therefore, the downstream part of the synthetic pathway was less affected for more complicated *N*-glycans such as Gal2F2GN2M3FX. These results may provide evidence that the intermediate complex type *N*-glycans play an important role in plants under excessive stress conditions.

**Table 1:** Estimated *N*-glycan structures obtained from peaks E1, E2, E3, and M1.

Peak	Structure	Abbreviation	Ratio	
			Control	SNCs exposure
E1		^GN^M3FX	3 (0.07)	15 (0.02)
E2		_GN_M3FX	5 (0.06)	24 (0.01)
E3		GN2M3X	3 (0.04)	18 (0.01)
M1		Gal2F2GN2M3FX	18 (0.02)	60 (0.00)

Standard errors are in parentheses.

Each *N*-glycan was also expressed in terms of percentage proportion relative to the GN2M3FX structure.

### Free-*N*-glycan analysis of SNC exposed *O. sativa* roots

**Figure 4:**
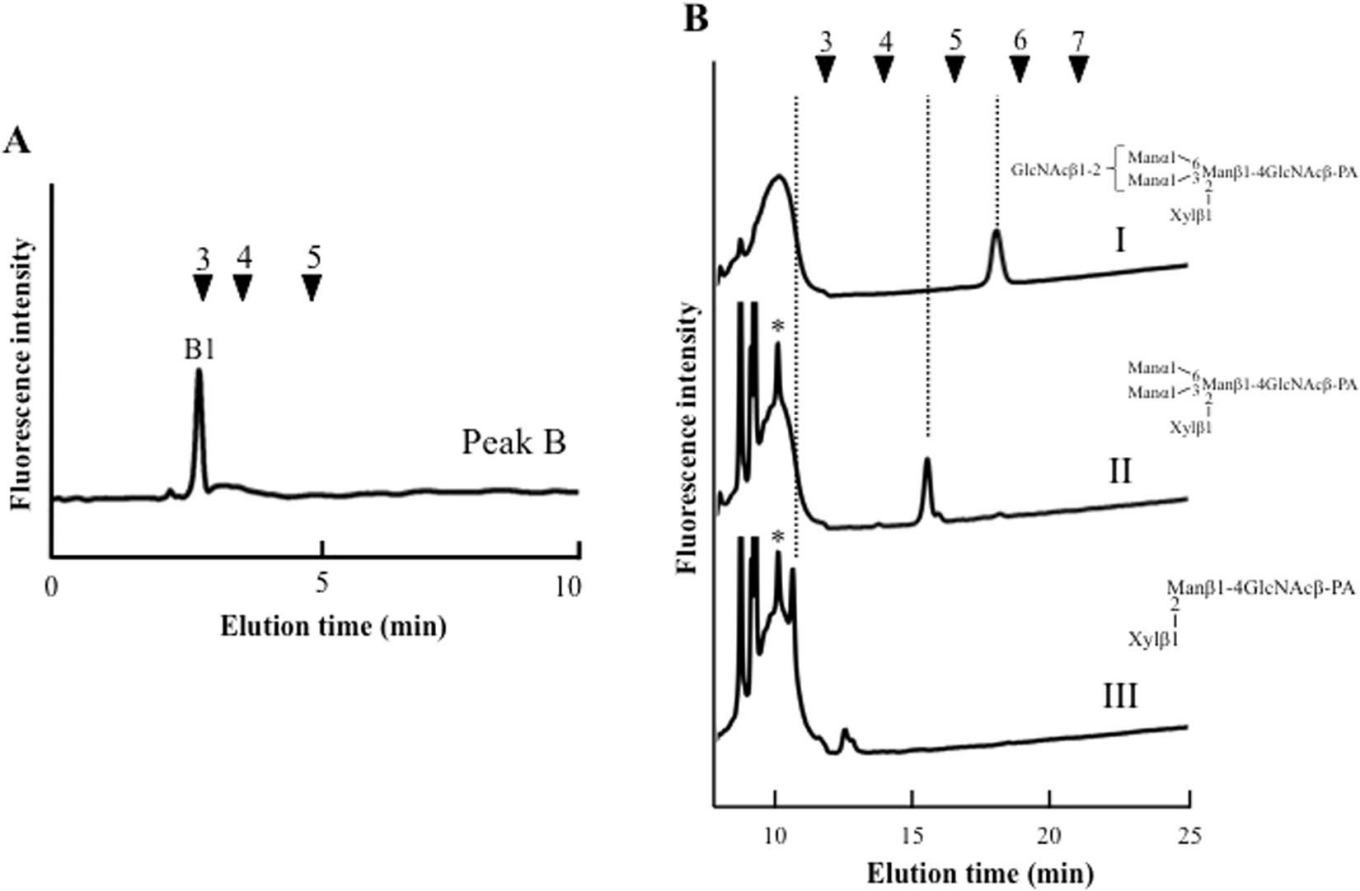
Structural analysis of peak B1. (**A**) Result of second reversed phase HPLC analysis of peak B1. (**B**) Sequential enzyme digestions of peak B1, I: peak B1, II: beta-*N*-acetylhexosaminidase digestion of I, III: alpha-mannosidase digestion of II. The peaks marked by the asterisks indicated non-specific peaks.

Reversed phase HPLC analysis revealed that peak B1 were eluted at around 3 min; therefore, to purify peak B1, twice reversed phase HPLC was performed (Fig. 4A). Peak B1 was further analyzed using MALDI-TOF mass spectrometry and sequential enzyme digestion. Mass spectrometry analysis of peak B1 showed that the *m/z* ratio was 1143.90 (Na^+^), which corresponded to (Hex)_3_(HexNAc)_2_(Pent)_1_-PA. The *m/z* ratio and elution position on reversed phase HPLC revealed that the *N*-glycan structure of peak B1 was predicted to be a free-GNM3X structure [GlcNAc_1_Man_3_Xyl_1_GlcNAc_1_-PA] with one GlcNAc residue at the reducing end. To ascertain the *N*-glycan structure of peak B1, peak B1 was enzymatically digested with two exoglycosidases. Peak B1 [GlcNAc_1_Man_3_Xyl_1_GlcNAc_1_-PA] was converted to Man_3_Xyl_1_GlcNAc_1_-PA, releasing one GlcNAc residue by beta-*N*-acetylhexosaminidase (Fig. 4B-II), and Man_3_Xyl_1_GlcNAc_1_-PA was further converted to Man_1_Xyl_1_GlcNAc_1_-PA, releasing two mannose residues by alpha-mannosidase (Fig. 4B-III). Thus, peak B1 was assigned as free-GNM3X structure with one GlcNAc residue at the reducing end (Fig. 4B-I).

**Figure 5:**
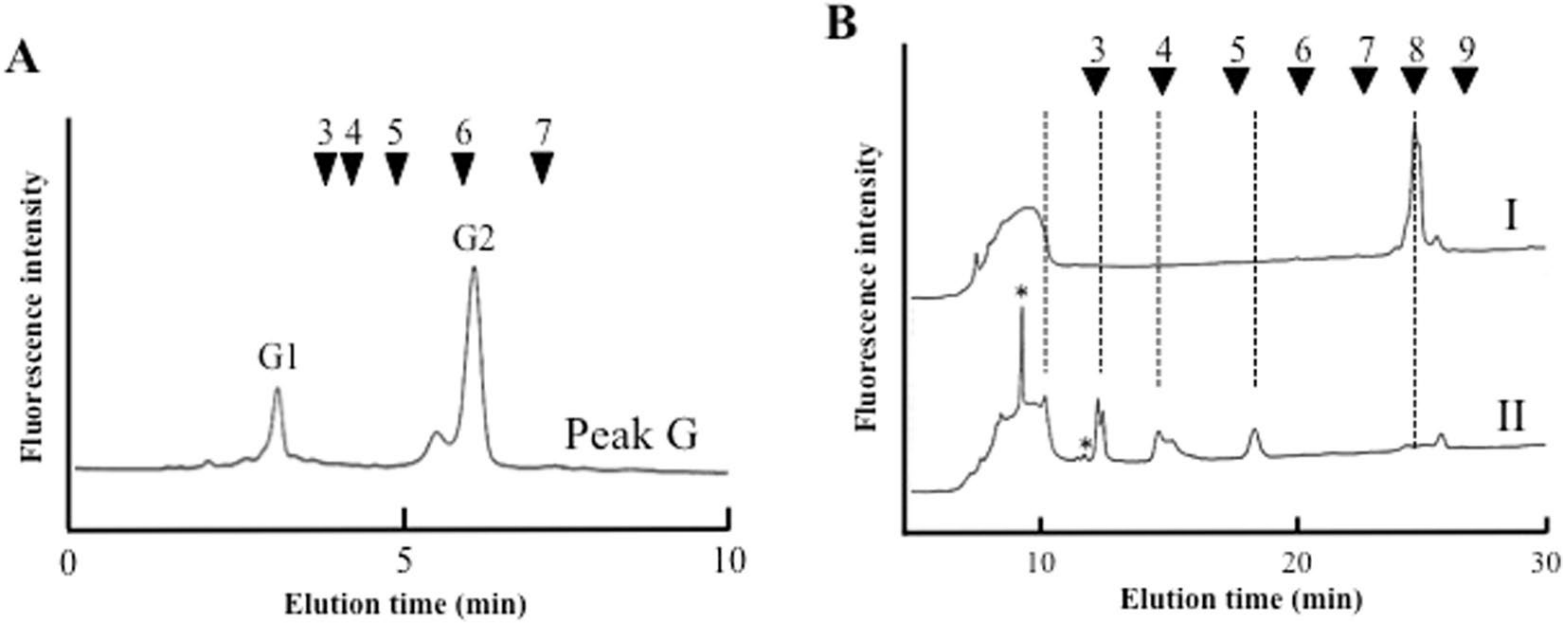
Structural analysis of peak G1. (**A**) Result of reversed phase HPLC analysis of peak G. (**B**) Exoglycosidase digestion of peak G1, I: peak G1, II: alpha-mannosidase digestion of I. The peaks marked by the asterisks indicated non-specific peaks.

Peak G is a major component of *O. sativa* roots (Fig. 2). As a result of reversed phase HPLC, two major peaks were detected; as a result of *N*-glycan two-dimensional mapping, the retention time of peak G2 corresponded to that of known position *N*-glycan, GN2M3FX^20^, and the other one was eluted at around 3 min (Fig. 5A, peak G1). In a similar way to peak B1, peak G1 was analyzed using MALDI-TOF mass spectrometry and exoglycosidase digestion. Mass spectrometry analysis showed that the *m/z* ratio was 1456.67 (Na^+^), which corresponded to (Hex)_7_ (HexNAc)_1_-PA. The mass value and elution position of reversed phase HPLC revealed that peak G1 was predicted to be a free-M7 structure [Man_7_GlcNAc_1_-PA]. To confirm the *N*-glycan structure, peak G1 was enzymatically digested with alpha-mannosidase. As a result, peak G1 [Man_7_GlcNAc_1_-PA] was converted to Man_4_GlcNAc_1_-PA, Man_3_GlcNAc_1_-PA, Man_2_GlcNAc_1_-PA, and Man_1_GlcNAc_1_-PA, releasing from three to six mannose residues by alpha-mannosidase (Fig. 5B-II); therefore, peak G1 was assigned as free-M7 structure.

As shown in Table 2, the proportion of free-GNM3X (peak B1) increased six fold after SNC exposure. In contrast to free-GNM3X, free-M7 increased three fold after SNC exposure (Table 2). To date, high-mannose type free-*N*-glycans have been detected in plant during development^21,22^, and other complex type free-*N*-glycans have been detected in the culture broth of rice cultured cells^23^ and *Egeria densa*^24^, and most of them have Lewis a structure [Gal beta1-3(Fuc alpha1-4)GlcNAc beta1-] that is characteristic of the *N*-glycan of extracellular glycoproteins. Maeda *et al*. discussed that a mechanism responsible for the production of complex type free-*N*-glycans is present under special or artificial conditions and native plant tissues^24^. In animal cells, the accumulation of sialyl free-*N*-glycans is caused by a decline in free-*N*-glycan metabolism by basal autophagy^25^. Mkhikian *et al*. demonstrated that alternative *N*-glycan structures were generated under unusual growth conditions^26^. These observations suggest that the occurrence of specific *N*-glycan structures is involved in cell conditions under excessive stress conditions.

**Table 2:** Proposed free-*N*-glycan structures.

**Peak**	**Structure**	**Abbreviation**	**Ratio**	
			Control	SNCs exposure
B1		Free-GNM3X	9 (0.00)	58 (0.00)
G1		Free-M7	16 (0.02)	45 (0.06)

Standard errors are in parentheses.

The elongation of *O. sativa* shoots was unaffected by SNC exposure (Fig. 1A), and *N*-glycan structures were also unaffected. We suggested that a root defense mechanism serves to protect shoot development from SNC toxicity, and *O. sativa* roots may have a defense mechanism against soil environmental changes. Although the generation of high-mannose type free-*N*-glycans is generally caused by the deglycosylation from misfolded glycoproteins in ER-associated degradation system^27^, there is almost no information about the biological significance of complex type free-*N*-glycan. Our results showed that the increase of complex type free-*N*-glycans was caused by the effect of SNCs, suggesting that complex type free-*N*-glycan itself in *O. sativa* roots has an important role for resistance mechanism against excessive environmental changes. *N*-Glycan is one of the post-translational modifications, and the structure and quantity are sensitive to ambient environment. In addition, *N*-glycans are generated by the result of the integrated multiple gene expression, and the biosynthesis is strictly controlled by many glycosyltransferases and glycosidases in each specific organelle. Though, it is difficult to identify which genes are specifically affected under stress condition, *N*-glycan analysis can derive the related genes and synthetic regions. Therefore, *N*-glycan analysis can predict to the initial response to environmental changes at comprehensively, and the transition is valuable for studying the relationship between environmental changes and biological response. As the case for monitoring the signaling pathway of plant under environmental changes, novel plant nanobionics approach has been reported^28^. Our results revealed a correlation between free-*N*-glycans and plant development under excessive stress conditions and also demonstrated that free-*N*-glycan transitions are valuable as stress markers for assessing trace environmental changes. This is the first report of the relationship between “environmental changes and glycome transition”, and the present study originally adapted glycome transition to environmental toxicology and proposed a new category called “Environmental glycobiology”.

## Materials and Methods

### Chemicals

Purified SNCs (28.4 ± 8.5 nm, release 81.1% Ag^+^ suspended in distilled water^29^) were purchased from Utopia Silver Supplements (Utopia, TX, USA). Cosmosil packed columns, 5NH_2_-MS (4.6 ID × 150 mm) and 5C_18_-P (4.6 ID × 150 mm), were purchased from Nacalai Tesque (Kyoto, Japan). Known-position PA-sugar chains were purchased from Takara Bio Inc. (Shiga, Japan). alpha-Mannosidase (from jack bean) was purchased from Sigma (MO, USA). beta-*N*-acetylhexosaminidase (from jack bean) was purchased from Prozyme (CA, USA).

### Plant materials and sample preparation

Seeds of *O. sativa* (Koshihikari) were supplied by Itakura Agricultural Cooperative Society, Japan. Before each experiment, seeds were washed five times with water and then washed twice with deionized water. The washed twelve seeds were then placed in a plate and immersed in 7 mm depth of water (control) or each concentration of SNCs suspension (0.5, 1.0, 1.5, 3.0, 5.0, 10.0, and 25.0 mg/L of SNCs). The plates were placed in a controlled environmental chamber, and kept at 37 °C for 96 h in the dark. The resulting seedlings were washed and then dried.

### Preparation of pyridylaminated *N*-glycans from *O. sativa* seedlings

*N*-Glycan preparation was performed according to the method of Natsuka *et al*.^30^. Dried shoots and roots were ground in a mortar at room temperature, and a ten milligram sample was used. *N*-Glycans were prepared by hydrazinolysis, *N*-acetylation. The reducing ends of the liberated *N*-glycans were then tagged with a fluorophore, 2-aminopyridyne (pyridylaminated *N*-glycans; PA-*N*-glycans), as described in previous paper^20^. These preparations were performed following details in Hase *et al*.^31^ with minor modifications.

### Separation of PA-*N*-glycans

Size-fractionation HPLC was performed in a Cosmosil 5NH_2_-MS column (4.6 ID × 150 mm) at a flow rate of 0.8 mL/min at 40 °C. PA-*N*-glycans were detected with a fluorescence spectrophotometer at 310 nm excitation and 380 nm emission. Reversed phase HPLC was performed on a Cosmosil 5C_18_-P column (4.6 ID × 150 mm) at a flow rate of 1.5 mL/min at 40 °C. The detection of PA-*N*-glycans performed by use of fluorescence spectrophotometer at 315 nm excitation and 400 nm emission. Each HPLC conditions were described in previous paper^20^.

### Mass spectrometry analysis of PA-glycans

MALDI-TOF mass spectrometry analysis was then performed using an AXIMA resonance instrument (Shimadzu) in reflector mode. Sample preparation was described in previous paper^20^.

### Glycosidase digestion of PA-*N*-glycans

A two picomoles of PA-*N*-glycans was prepared in 1 microL of accessory reaction buffer (5 mM CaCl_2_, 10 mM ammonium acetate buffer, pH 4.5) and 2 microL of D. D. W, 1 microL of beta-*N-*acetylhexosaminidase (0.05 units/microL) was added, and the mixture was incubated at 37 °C for 4 h, and then 1 microL of jack bean alpha-mannosidase (19 units/mg) and 5 microL of 10 mM ammonium acetate buffer (pH 4.5) were added, and the mixture was incubated at 37 °C for 1 h. alpha-Mannosidase digestion was described in previous paper^20^. To stop all reactions, the mixtures were boiled for 5 min at 95 °C, and the mixture was analyzed by size-fractionation HPLC.

## Electronic supplementary material

Supplementary Information 
