# Supplementary material for: Effects of silver nanocolloids on plant complex type N-glycans in Oryza sativa roots
Source: Sci Rep. 2018 Jan 17;8:1000. doi: 10.1038/s41598-018-19474-z (PMC5772479; doi:10.1038/s41598-018-19474-z)
Supplement: Supplementary file 1 — Supplementary Information [file 41598_2018_19474_MOESM1_ESM.pdf]

Effects of silver nanocolloids on plant complex type *N*-glycans in *Oryza sativa* roots

Risa Horiuchi[a], Yukari Nakajima[b], Shosaku Kashiwada[a,c], *Nobumitsu Miyanishi[a,b,c,d]

[a] Graduate School of Life Sciences, Toyo University, Gunma, 374-0193, Japan
[b] Department of Life Sciences, Toyo University, Gunma, 374-0193, Japan
[c] Research Centre for Life and Environmental Sciences, Toyo University, Gunma, 374-0193, Japan
[d] Graduate School of Food and Nutritional Sciences, Toyo University, Gunma, 374-0193, Japan

* Correspondence and requests for materials should be addressed to Nobumitsu Miyanishi (email: miyanishi@toyo.jp)

Supplementary Figure

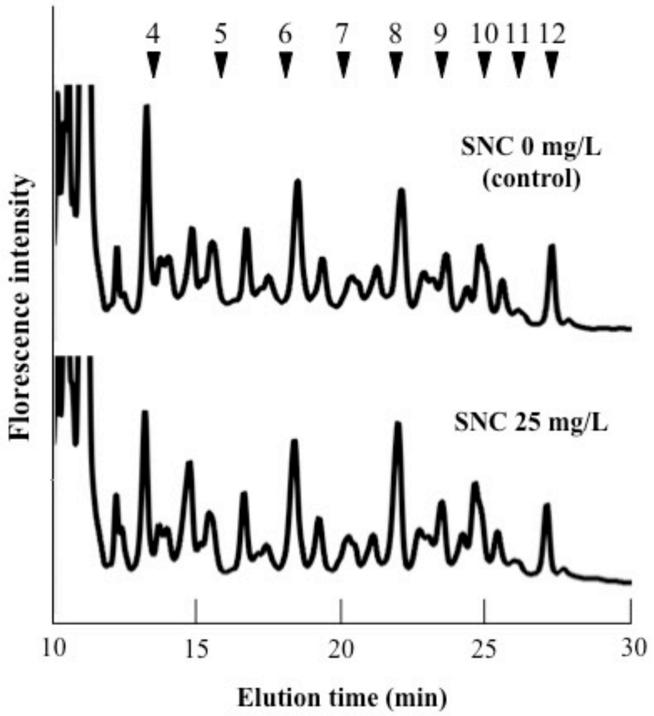

Supplementary Figure 1.

Results of size-fractionation HPLC analysis of *N*-glycans derived from *O. sativa* germs after 48 h incubation.
(I) Control, (II) SNCs exposure. Arrowheads 4-12 indicate the degree of polymerization of PA-isomaltooligomer.
